# 

**Published:** 1883-12

**Authors:** 


### CONTENTS:

Original Communications: PAGE.

I. The Treatment of Bright's Disease. By Charles W. Purdy, M.D. ........ 561

II. Human Parasites. By F. W. Goding, M.D. ........ 587

III. Pre-Natal Influences as Affecting the Teeth. By Geo. H. Cushing, D.D.S. 610

Domestic Correspondence:

IV. Cincinnati Letter ........ 616

Society Reports:

V. Chicago Medical Society ........ 623

Chicago Pathological Society ........ 627

Michigan State Board of Health ........ 629

Illinois State Board of Public Charities, Report of F. H. Wines, Secretary ........ 631

Editorial:

The Intervention of the State in Medical Affairs ........ 640

Selections: PAGE.

A Lecture on the Tubercle-Bacillus and Phthisis. By T. Henry Green, M.D., F.R.C.P. ........ 647

The Frequent Repetition of Doses By John W. Martin, M.D. ........ 655

Two Cases of Poisoning with Illuminating Gas Successfully Treated by the Inhalation of Oxygen. By Alonzo Clark, M.D., LL.D. ........ 657

Diseases of Children—Palatable Drugs for Children. By Frederick Churchill, M.D., F.R.C.S. ........ 659

Obituary:

Dr Frank L. Rea ........ 663

Books Received ........ 666

Items ........ ...615, 622, 639, 645, 646, 662, 667

